# Prevalence of Metabolic-Dysfunction-Associated Steatotic Liver Disease in Patients with Psoriasis and Psoriatic Arthritis

**DOI:** 10.3390/diagnostics16142292

**Published:** 2026-07-22

**Authors:** Nuria Vegas-Revenga, Cristina San Juan López, Blanca Sampedro Andrada, Victoria Morillo Montañés, Irati Urionaguena-Onaindia, Nahia Plaza-Aulestia, Sandra Pérez Prado, Itziar Calvo Zorrilla, Oihane Ibarguengoitia-Barrena, David Montero Seisdedos, Libe Ibarrola-Paino, Lucía Vega-Álvarez, Aitor Orive Calzada, José Francisco Garcia Llorente

**Affiliations:** 1 Department of Rheumatology, Hospital Universitario Galdakao-Usansolo, 48960 Galdakao, Bizkaia, Spain; 2 Biobizkaia Health Research Institute, Osakidetza, Cruces, 48903 Barakaldo, Bizkaia, Spain; 3 Department of Medicine, Euskal Herriko Unibertsitatea, 48940 Leioa, Bizkaia, Spain; 4 Department of Gastroenterology and Hepatology, Hospital Universitario Galdakao-Usansolo, 48960 Galdakao, Bizkaia, Spain; 5 Centro Vasco de Aparato Digestivo, 48014 Bilbao, Bizkaia, Spain; 6 Department of Dermatology, Hospital Universitario Galdakao-Usansolo, 48960 Galdakao, Bizkaia, Spain

**Keywords:** psoriatic disease, metabolic-dysfunction-associated steatotic liver disease, fibrosis, steatosis, disease-modifying antirheumatic drugs, metabolic syndrome

## Abstract

**Background/Objectives:** Psoriatic disease (PsD) is a chronic cutaneous and systemic inflammatory condition associated with an increased risk of metabolic-dysfunction-associated steatotic liver disease (MASLD) and its complications. Our objective was to determine the prevalence of MASLD in our overall cohort and to analyze it across PsO, PsA, and healthy control groups. The secondary objective was to describe hepatic steatosis and fibrosis using transient elastography (FibroScan^®^) and to characterize hepatic scores within each study group. The third objective was to assess whether specific cardiovascular risk factors were associated with the development of hepatic steatosis or fibrosis in individuals with PsD and healthy controls. **Methods:** A cross-sectional study was conducted at a single tertiary care center, enrolling consecutive patients from the Dermatology and Rheumatology Departments. Individuals with pre-existing liver disorders were excluded. The control cohort comprised 102 healthy volunteers without chronic inflammatory or hepatic conditions. All participants underwent comprehensive clinical assessments, serum biomarker analysis, and FibroScan^®^. **Results:** A total of 330 participants were included, 228 with PsD (101 with psoriasis (PsO) and 127 with psoriatic arthritis (PsA)) and 102 healthy controls. MASLD was present in 39% of the overall cohort, with a prevalence of 47.5% in PsO, 32.2% in PsA, and 8.8% in healthy controls (*p* < 0.001). FibroScan^®^ measurements indicated a tendency toward increased liver stiffness and higher controlled attenuation parameter (CAP) values in patients with PsD. Both PsO and PsA groups showed a higher prevalence of cardiometabolic risk factors compared with controls (*p* < 0.05). Non-invasive fibrosis indices were also significantly altered in both patient groups. **Conclusions:** Patients with PsD demonstrated a higher prevalence of MASLD, hepatic fibrosis, and cardiometabolic abnormalities compared with healthy controls in our cohort.

## 1. Introduction

Psoriatic disease (PsD) is a chronic, immune-mediated inflammatory disorder influenced by genetic, environmental, and immunological factors. The worldwide prevalence of PsD is 0.9–8.5% [1]. Psoriatic arthritis (PsA) is a seronegative, chronic inflammatory arthropathy frequently associated with cutaneous psoriasis (PsO), with an estimated prevalence of 112 per 100,000 adults [1,2,3]. The diagnosis of PsA is sometimes challenging due to its substantial clinical and radiological heterogeneity, encompassing six domains of involvement: peripheral joints, axial skeleton, skin psoriasis, nail psoriasis, enthesitis, and dactylitis. Treatment strategies for PsO and PsA vary according to disease severity and clinical presentation, and may include topical agents, conventional disease-modifying antirheumatic drugs (DMARDs), or biologic therapies [2,3].

PsD is associated with a broad spectrum of comorbidities—including cardiovascular, metabolic, gastrointestinal, hepatic, and renal diseases—as well as an increased risk of gastrointestinal and other malignancies [4,5,6,7,8]. Patients with PsD also show a higher prevalence of metabolic-dysfunction-associated steatotic liver disease (MASLD) [7]. MASLD is defined by the presence of hepatic steatosis in conjunction with at least one cardiovascular risk (insulin resistance, abdominal obesity, atherogenic dyslipidemia, or hypertension) in the absence of clinically significant alcohol consumption or other secondary causes of steatosis [9].

MASLD, previously known as non-alcoholic fatty liver disease (NAFLD) [9], is considered the hepatic manifestation of metabolic syndrome (MetS)—a condition to which PsD is intrinsically linked. Although the co-occurrence of PsD and MASLD is not unexpected, growing evidence suggests that they share overlapping pathophysiological mechanisms, including systemic inflammation, insulin resistance, and impaired lipid metabolism, which may contribute to disease progression in both conditions [7]. MASLD is currently the most prevalent hepatic disorder [10]. Data from the US National Health and Nutrition Examination Survey (NHANES) using transient elastography indicate that MASLD affects approximately 25–30% of adults in the United States [11]. The burden is even higher among individuals with obesity and type 2 diabetes, where prevalence rates can exceed 70% [11]. PsD patients not only have a higher likelihood of developing MASLD, but they also face a greater risk of experiencing more advanced and clinically severe forms of the disease [7].

Therefore, patients with PsD and metabolic syndrome should be routinely screened for MASLD. Moreover, therapeutic decision-making should always account for potential hepatic risks, particularly in patients with MASLD who have evidence of fibrosis [10].

While various studies have been performed on the association of PsO with different comorbidities, including metabolic syndrome and MASLD [1,4,6,12,13,14,15,16,17,18], data in PsA are lacking. Only a few studies compared the prevalence of MetS between PsO and PsA, with contradictory results [19,20]. Furthermore, no studies specifically focused on metabolic liver disease in PsO compared with PsA and healthy controls. Taking these considerations into account, the primary objective of this study was to determine the prevalence of MASLD in our overall cohort and to analyze it across the PsO, PsA, and healthy control groups at a tertiary-care hospital. The secondary objective was to describe hepatic steatosis and fibrosis using transient elastography (FibroScan^®^) and to characterize hepatic scores within each study group. The third objective was to assess whether specific cardiovascular risk factors were associated with the development of hepatic steatosis or fibrosis in individuals with PsD and healthy controls.

## 2. Materials and Methods

### 2.1. Study Design and Population

This observational study aimed to assess the prevalence of MASLD and hepatic fibrosis assessed by FibroScan^®^ Compact 530 (Echosens, Paris, France) among patients over 18 years diagnosed with either PsO or PsA, who were diagnosed and followed by the dermatology or rheumatology departments, respectively, at the Hospital Universitario Galdakao-Usansolo (Vizcaya, Spain). Patients with any severity or duration of inflammatory disease were eligible for inclusion. None of the participants enrolled in the study had a known history of liver disease. Patients were enrolled consecutively during outpatient consultations over a year and were compared with a control group of healthy volunteers.

The control group was consecutively enrolled and consisted of volunteers, most of whom were hospital staff or relatives and acquaintances of enrolled patients.

The present study was conducted in accordance with the Declaration of Helsinki. The local ethics committee approved the research protocol and informed consent was obtained from all participants.

### 2.2. Participants and Data Collection

After obtaining informed consent, participants completed a comprehensive baseline questionnaire that captured detailed clinical and lifestyle information. Individuals with a history of chronic liver disease—including viral hepatitis B or C, autoimmune hepatitis, monogenic, other steatotic liver diseases or previously documented alcohol-related liver injury—were excluded. To minimize confounding, we also excluded participants with current use of medications known to cause hepatotoxicity or with prior drug-induced liver injury, except for therapies routinely used in the management of PsO or PsA. Alcohol intake was recorded using standardized units, and participants exceeding accepted thresholds for low-risk consumption (>30 g/day for men and >20 g/day for women) were not eligible for inclusion. The questionnaire further documented clinical and metabolic comorbidities such as body mass index (BMI), type 2 diabetes, dyslipidemia, and hypertension, which were considered key inclusion variables given their relevance to MASLD.

Clinical records related to PsO and PsA were reviewed, including time since diagnosis and treatment history. A member of the dermatology department assessed each participant to calculate the Psoriasis Area and Severity Index (PASI) score at the time of enrollment. PASI is a validated tool that quantifies the average redness, thickness, and scaliness of psoriatic lesions, weighted by the extent of body surface area affected, and is commonly used to assess disease severity in clinical trials [21,22].

A rheumatologist evaluated whether each patient with PsA exhibited exclusively peripheral manifestations (Classification Criteria for Psoriatic Arthritis (CASPAR)) [23] or a mixed clinical pattern (axial and peripheral), and determined the disease activity status at the time of study inclusion. Disease activity was assessed through the disease activity index (DAPSA) for the articular involvement.

The control group completed a baseline questionnaire designed to gather comprehensive information on medical history, metabolic risk factors, current pharmacological treatments, and both current and past alcohol consumption. None of the participants in the control group had a documented history of liver disease or autoimmune conditions.

### 2.3. Clinical and Biochemical Data

Patients who met the inclusion criteria underwent a fasting blood draw, and liver elastography with controlled attenuation parameter (CAP) measurement. Anthropometric measurements, including body weight and height, were also obtained in order to calculate BMI. BMI was classified as [24]: normal weight (BMI < 25 kg/m^2^), overweight (BMI 25–30 kg/m^2^), and obese (BMI ≥ 30 kg/m^2^). Obesity was divided into grade I (30–35 kg/m^2^), II (35–40 kg/m^2^) and III (>40 kg/m^2^).

Blood tests included liver-associated enzymes (alanine aminotransferase [ALT] and aspartate aminotransferase [AST]), glucose, glycated hemoglobin (HbA1c), homeostasis model assessment of insulin resistance (HOMA), insulin levels, and a lipid profile comprising total cholesterol, high-density lipoprotein cholesterol (HDL-C), low-density lipoprotein cholesterol (LDL-C), and triglycerides. Additional parameters measured were blood urea nitrogen (BUN), creatinine, C-reactive protein (CRP), and erythrocyte sedimentation rate (ESR).

Transient elastography was performed in all participants during routine clinical appointments, and the staff were not aware that the patients were enrolled in a study. The examination was conducted using a FibroScan^®^ Compact 530 device (Echosens, Paris, France). Measurements were obtained from the right hepatic lobe through the intercostal spaces, with the patient in supine position and the right arm maximally abducted. The transducer probe, covered with coupling gel, was positioned between the ribs overlying the right hepatic lobe, and the operator identified a liver region at least 6 cm thick and free of large vascular structures. Transient elastography was always initiated with the M probe; if no valid measurements were obtained, the assessment was repeated using the XL probe. Once the target area was located, the probe button was activated to acquire measurements. A minimum of ten valid measurements were required for each participant, and results were considered reliable when the interquartile range-to-median ratio (IQR/M) was ≤30%. Liver stiffness was expressed in kilopascals (kPa), with the median value representing the liver’s elastic modulus. Controlled attenuation parameter (CAP) values were simultaneously recorded to quantify hepatic steatosis.

For fibrosis assessment, significant fibrosis was defined as ≥F2 (liver stiffness measurement [LSM] > 7.5 kPa). This threshold is supported by previously validated transient elastography studies. In particular, Castera et al. reported that liver stiffness values above 7.0 kPa are indicative of significant fibrosis (F2), while values between 2.5 and 7.0 kPa are generally associated with absent or mild fibrosis (F0–F1) [25]. For steatosis assessment, significant steatosis was defined as ≥S1 (CAP > 240 dB/m). Although CAP thresholds vary across studies depending on the population analyzed and the statistical methodology applied, published validation studies and meta-analyses have reported cut-off values for mild steatosis (S ≥ 1) within a similar range [26].

Scores were calculated utilizing the fibrosis-4 (FIB-4) index, the aspartate aminotransferase-to-platelet ratio index (APRI), the hepamet fibrosis score (HFS), and the NAFLD score (NFS). The FIB-4 index is derived from age, aminotransferase levels and platelet count, and is widely used to estimate the probability of advanced fibrosis [27]. The APRI is based on the ratio of AST to platelet count, serving as a simple surrogate marker of hepatic fibrosis [28]. The HFS incorporates demographic, biochemical and metabolic variables to provide a validated prediction of advanced fibrosis in MASLD [29]. The NFS combines age, BMI, glycemic status, aminotransferase ratio, platelet count and albumin concentration, and is employed to stratify the risk of advanced fibrosis in patients with MASLD [29].

### 2.4. Statistical Analysis

This observational study compared the prevalence of MASLD across three groups: patients with PsO, patients with PsA, and healthy controls. Quantitative variables were expressed as mean ± standard deviation (SD) when normally distributed and as median (interquartile range) when non-normally distributed. Continuous variables were compared among the three study groups using one-way analysis of variance (ANOVA). When the overall ANOVA was significant, post hoc pairwise comparisons were performed using Tukey’s honestly significant difference (HSD) test. Homogeneity of variances was assessed using Levene’s test. Categorical variables were compared using Pearson’s chi-square test or Fisher’s exact test when appropriate. A two-sided *p* value < 0.05 was considered statistically significant. Linear regression was used for multivariable analysis. All statistical analyses were performed using IBM SPSS 20 Statistics software (Armonk, NY, USA).

## 3. Results

### 3.1. The Characteristics of Patients

A total of 330 patients were enrolled in the study, 228 patients with PsD (101 patients with PsO and 127 with PsA) and 102 healthy volunteers. The mean age of our patients in the PsO group was 57.8 ± 12.7 years and in the PsA group was 59.7 ± 12.2 years. Table 1 presents the demographic and clinical characteristics of the study cohorts. Sex distribution differed significantly across groups, with a predominance of males in the PsO cohort (63.4%) and females in the control group (72.5%) (*p* < 0.001). The prevalence of diabetes mellitus was higher in PsO (13.9%) and PsA (12.6%) compared with controls (2.9%) (*p* = 0.016). Mean fasting glycemia did not differ significantly between groups (*p* = 0.067). Hypertension was observed in 44.6% of PsO patients and 29.1% of PsA patients, compared with 6.9% of controls (*p* < 0.001). Dyslipidemia was present in 44.6% and 44.9% of PsO and PsA patients, respectively, versus 7.8% of controls (*p* < 0.001). LDL-cholesterol levels were modestly but significantly higher in PsA (123.3 ± 34.3 mg/dL) compared with PsO (116.3 ± 33.9 mg/dL) and controls (119.9 ± 34.5 mg/dL) (*p* = 0.047). Triglyceride concentrations were significantly elevated in both patient groups compared with controls (*p* < 0.001). No differences were observed in the prevalence of hyperuricemia (*p* = 0.643). Moderate alcohol consumption was reported in men < 60 g/day and <40 g/day in women. ALT levels were slightly but significantly higher in both groups of patients compared with controls (*p* < 0.001), whereas AST levels were markedly elevated in PsO and PsA relative to controls (*p* = 0.015).

Regarding PsD characteristics, patients with exclusively cutaneous involvement (98 patients, 97%) had a mean disease duration of 10.5 ± 6.5 years. The mean PASI score in patients with PsO was 5.86 ± 6.8, while in the PsA subgroup it was lower (3.67 ± 3.6). Because of the limited sample size, further stratification by PASI categories was not feasible. When both groups were analyzed together, the overall mean PASI score was 3.5 ± 5.6.

Patients with PsA had a mean disease duration of 9.69 ± 6.33 years. Within the PsA subgroup, 28 (22.4%) patients presented axial and peripheral arthritis, whereas 95 (76%) exhibited only peripheral manifestations. The joint count assessment showed that 82.4% of patients had 0 tender joints and 86.4% had 0 swollen joints. More than 80% of patients with PsA were in clinical remission at the time of inclusion in the study.

Regarding biologic therapies, patients with PsO had received the following treatments: 21 patients had been treated with an anti-tumor necrosis factor (anti-TNF, 97% with a single agent and 3% with two different anti-TNF agent). All patients had been exposed to anti- interleukin-17 (anti-IL-17) therapy (98% to a single agent and 2% to more than one anti-IL-17 agent). In addition, 40 patients (39.6%) had received a Janus kinases (JAK) inhibitor, and 60.4% had been treated with an IL-12/23 inhibitor.

Among patients with PsA, 40 individuals (31.5%) had received an anti-TNF agent (89% one agent, 9.4% two agents, and 1.6% up to three different anti-TNF agent). A total of 121 patients had been treated with an anti-IL-17 therapy (95.3% with one agent and 4.7% with two agents). Furthermore, 8 patients had received a JAK inhibitor, and 9 patients had been treated with an IL-12/23 inhibitor.

### 3.2. Prevalence of MASLD in the Study Group

**Figure 1:**
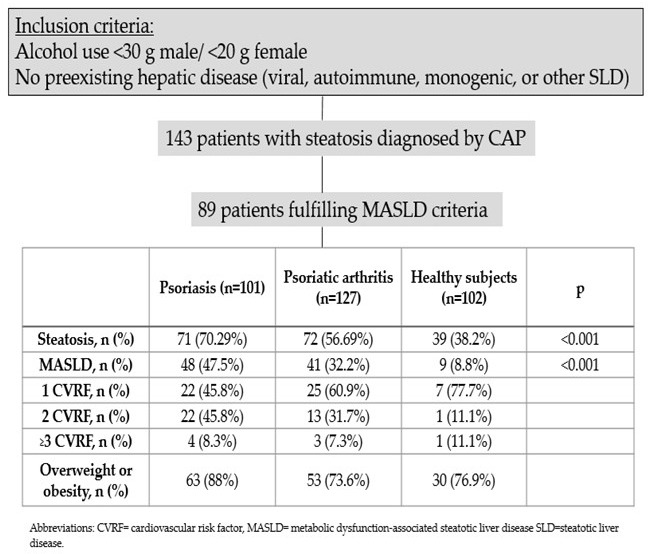
Flowchart illustrating patient selection and MASLD prevalence classification across psoriasis, psoriatic arthritis, and control cohorts. Abbreviations: CVRF = cardiovascular risk factor, MASLD = metabolic-dysfunction-associated steatotic liver disease, SLD = steatotic liver disease.

Figure 1 summarizes the prevalence of MASLD in the study population, which reached 39% overall. Upon stratification by cohort, MASLD was observed in 47.5% of PsO patients, 32.2% of PsA patients, and 8.8% of healthy controls. Most participants exhibited between one and three cardiovascular risk factors; fewer than 10% of PsO and PsA patients presented three or more, whereas this proportion was slightly higher among healthy controls (11.1%). Overweight or obesity status was highly prevalent across groups, affecting 88% of PsO patients, 73.6% of PsA patients, and 76.9% of controls.

### 3.3. Results of the Fibroscan and Scoring Tools

Table 2 summarizes FibroScan^®^ findings and non-invasive scoring tools. Mean liver stiffness was significantly higher in PsO (6.01 ± 3.4 kPa) compared with PsA (5.1 ± 1.7 kPa) and controls (4.9 ± 2.08 kPa) (*p* = 0.006). Advanced fibrosis (F3–F4) was more frequent in PsO (9.9%) than in PsA (6.3%) or controls (5.9%). CAP values were significantly increased in both patient groups, with PsO showing the highest mean (270.5 ± 53.7 dB/m) compared with PsA (257 ± 52.5 dB/m) and controls (229 ± 47.5 dB/m) (*p* < 0.001). Severe steatosis (S3) was observed in 32.7% of PsO and 25.2% of PsA patients, compared with 11.8% of controls. FIB-4 scores were significantly higher in PsD groups compared with controls (*p* = 0.024). NAFLD fibrosis scores shifted toward less negative values in both PsO and PsA cohorts, consistent with increased fibrosis risk (*p* < 0.001). APRI values did not differ significantly between groups (*p* = 0.075). HFS values were significantly elevated in PsO and PsA compared with controls (*p* = 0.006).

### 3.4. Cardiovascular Risk Factors Associated with Liver Steatosis and Fibrosis

In the multivariable analysis shown in Table 3, after adjustment for BMI, glycemia, LDL-cholesterol, and HDL-cholesterol, the study group remained independently associated with liver steatosis. Among the covariates included in the model, only BMI remained significantly associated with liver fibrosis (*p* = 0.004), whereas glycemia (*p* = 0.111), LDL-cholesterol (*p* = 0.387), and HDL-cholesterol (*p* = 0.505) were not independently associated with the outcome. Pairwise comparisons between study groups did not reach statistical significance after adjustment for multiple comparisons, although a trend toward significance was observed between PsO and PsA (CI −0.035–1.692; *p* = 0.064) for liver fibrosis. No differences were observed in liver steatosis. BMI remained independently associated with liver stiffness (B = 0.090, 95% CI 0.029–0.150; *p* = 0.004), whereas study group was not independently associated with liver stiffness after adjustment for metabolic covariates.

**Table 3:** Multivariable analysis of fibrosis after adjusting for cardiovascular risk factors.

Predictor	B	95% CI	*p*
PsO vs. HC	−0.985	−3.113 to 1.143	0.363
PsA vs. HC	−1.814	−3.932 to 0.304	0.093
BMI	0.090	0.029 to 0.150	0.004
Glycemia	0.013	−0.003 to 0.028	0.111
LDL-C	0.003	−0.004 to 0.011	0.387
HDL-C	0.007	−0.015 to 0.029	0.505

Abbreviations: BMI = body mass index, CI = confidence interval, HDL-C = high density lipoprotein-cholesterol, LDL-C = low density lipoprotein-cholesterol, PsO = psoriasis, PsA = psoriatic arthritis, HC= healthy controls.

## 4. Discussion

PsD is increasingly recognized as involving immunometabolic pathways that may contribute to metabolic comorbidities, including MASLD. Although shared inflammatory mechanisms are thought to play a role, both systemic inflammation and metabolic factors appear to influence hepatic involvement. Lifestyle habits and cumulative exposure to systemic therapies may also modulate hepatic risk, highlighting the need for coordinated assessment across dermatology, rheumatology, and hepatology.

The prevalence of MASLD in our cohort (39%) is consistent with previous reports in PsD, which have described rates ranging from 36% to 43% in similarly characterized populations [30,31]. Although methodological differences across studies—such as diagnostic thresholds, cohort composition, and the assessment of fibrosis—may account for some variability, the overall findings suggests that MASLD is a common comorbidity in PsD.

The low prevalence of MASLD observed in our healthy controls (8.8%) likely reflects the characteristics of this selected cohort. In contrast, the presence of hepatic steatosis (38.2%) is more consistent with rates reported in population-based screening programs [32]. These findings suggest that the recently adopted MASLD criteria may influence prevalence estimation by distinguishing steatosis detected through screening from MASLD defined by metabolic and cardiovascular risk components. Within this framework, our healthy cohort aligns with expected steatosis prevalence but shows a lower MASLD prevalence, likely due to their reduced cardiometabolic risk burden.

A bidirectional hepato-dermal axis has been proposed to link cutaneous inflammation with hepatic metabolic dysfunction. Keratinocytes and skin-derived lymphocytes produce cytokines—IL-6, IL-17, and TNF-α—that can circulate systemically, impair insulin signaling and promote hepatic steatosis [33]. Conversely, hepatic steatosis and inflammation may potentiate psoriatic activity, creating a self-reinforcing cycle that accelerates both dermatologic and hepatic disease progression [33]. This mechanistic interplay is consistent with epidemiological data showing a higher prevalence features consistent with steatotic liver disease among patients with PsO and an even greater burden among those with PsA or moderate-to-severe skin disease [13,16,18,34]. Ultrasound-based studies report MASLD prevalence rates from approximately 47% to 59% among mixed PsO cohorts [18,35], with biopsy-confirmed hepatic steatosis observed in a substantial proportion of patients [35]. To assess steatosis, ultrasound has traditionally been used, but one of the most important limitations has been that it is an operator dependent test. Karlas et al., in an individual patient-data meta-analysis, demonstrated the variability of CAP thresholds used for steatosis detection while validating CAP as a reliable non-invasive tool for steatosis assessment [26]. More recent studies, including that of Eddowes et al., have further confirmed the diagnostic utility of CAP in patients with MASLD. Consequently, the threshold of 240 dB/m adopted in our study falls within the range of validated values reported in the literature for the detection of at least mild hepatic steatosis [36].

Our findings align with these observations; we initially identified higher rates of steatosis and fibrosis in patients with PsD; however, after adjusting for common metabolic factors such as hypertension, dyslipidemia, and BMI, these differences were no longer statistically significant, although a tendency persisted, likely reflecting limited statistical potency. The small sample size and the composition of our healthy control group—predominantly premenopausal women with fewer cardiovascular risk factors—may have contributed to an apparent overestimation of disease impact in PsD. Although advanced fibrosis did not differ significantly between PsO and PsA groups, the numerically higher rates observed in PsO may require additional exploration. This tendency, while not statistically significant, could relate to differences in inflammatory burden, metabolic profile, treatment exposure, or the chronicity of cutaneous inflammation. Nonetheless, the modest sample size may have limited our ability to detect significant differences.

Multivariable analyses from previous studies indicate that MASLD may be associated with more severe cutaneous disease [8,37]. In one cohort [38], MASLD emerged as the only significant independent predictor of higher PASI score after adjustment for age, sex, BMI, disease duration and alcohol intake. Longitudinal registry data indicate that patients with PsO who require systemic therapy are at increased risk of developing liver disease, particularly MASLD and cirrhosis, compared both with matched controls and with patients with rheumatoid arthritis [39]. Notably, this association persisted in sensitivity analyses excluding methotrexate exposure, implying that intrinsic disease-related factors contribute to hepatic risk beyond the effects of specific hepatotoxic medications [39]. In our study, we could not confirm this association, likely due to the high prevalence of systemic treatment use and limited statistical power. Moreover, the cross-sectional design and the relatively small number of patients with high PASI scores may have reduced our ability to detect a robust relationship between skin severity and liver involvement. Several limitations of PASI are well recognized, including its subjectivity, complexity and the variability introduced by estimating the percentage of affected body surface area. In our cohort, PASI was assessed at the time of screening in patients with stable autoimmune disease. Although we observed numerically higher PASI scores in some subgroups, the sample size may have limited our ability to detect statistically significant differences, so larger cohorts may be needed to confirm this observation.

Metabolic comorbidities were markedly more common in PsD patients than in controls, consistent with the well-established association between PsD, insulin resistance, dyslipidemia, and obesity [40]. We found a higher prevalence of traditional cardiometabolic risk factors in multivariate analyses. An important limitation of our study is that healthy controls were predominantly women, whereas patients were more frequently men. Cardiometabolic factors are more prevalent among men compared to premenopausal women, and this could have been a confounding factor that must be taken into account. These healthy volunteers were also found among workers and relatives of hospital workers, who could be more aware of the importance of healthy habits, causing a selection bias. Despite these imbalances, in our multivariate analysis, BMI showed an independent association with liver stiffness in patients with PsD. The higher prevalence of overweight and obese individuals in this group could reflect a predisposition shaped by the underlying inflammatory milieu.

Conventional disease-modifying antirheumatic drugs have traditionally been associated with hepatic steatosis and fibrosis progression. Given the high cumulative exposure to biologic and targeted synthetic therapies in our cohort, it is important to consider their potential influence on hepatic inflammation and MASLD progression. Anti-TNF agents have been associated with reductions in systemic inflammation and improvements in insulin sensitivity, which may attenuate hepatic steatosis and inflammatory activity; TNF α blockade has also been shown to decrease hepatic necroinflammation and slow fibrosis progression in metabolic liver disease [41]. Evidence from inflammatory bowel disease further suggests that anti-TNF therapy is less frequently used among patients with fibrosis and is independently associated with lower odds of fibrosis [42].

IL-17 plays a central role in neutrophil recruitment, insulin resistance, steatohepatitis, and hepatic stellate cell activation. Blocking IL-17 improves fibrosis in MASLD [43,44]. Clinical data remain heterogeneous, but IL-17 and IL-23 inhibitors may exert hepatoprotective effects. Dual IL-17/IL-23 blockade (already effective in psoriasis) has been proposed as a promising strategy for MASLD phenotypes with high IL-17 activity [45]. In addition, real-world data from long-term cohorts of patients treated with IL-17 inhibitors show that cardiometabolic comorbidities and higher BMI are associated with lower rates of minimal disease activity and reduced drug retention, highlighting the influence of metabolic dysfunction on systemic inflammatory control [46]. Although these studies do not assess hepatic outcomes, they reinforce the interplay between metabolic burden, inflammatory activity, and MASLD risk.

JAK inhibitors, while effective for psoriatic disease, are known to alter lipid metabolism and increase LDL-C and HDL-C levels, although their direct impact on hepatic steatosis or fibrosis remains uncertain [47]. Experimental studies suggest that JAK inhibition may attenuate fibrosis progression and promote fibrosis reversal, but these findings have not yet been linked to psoriasis treatment [48].

Emerging evidence also indicates that newer biologic therapies may reduce hepatic steatosis and triglyceride accumulation in hepatocytes [49]. Overall, the extensive use of biologics in our PsO and PsA populations may have modulated hepatic inflammation and metabolic outcomes in PsD. Although biologic and targeted therapies were recorded, these variables were not incorporated into the main analysis because the study was not powered to evaluate treatment-specific effects, and treatment distribution across subgroups was heterogeneous. Moreover, the observational and cross-sectional design does not allow for temporal inferences regarding the impact of current or past therapies on liver outcomes. We acknowledge that current treatment, cumulative exposure, and prior methotrexate use could have acted as potential confounders. Future analyses incorporating therapeutic exposure could help clarify whether certain biologic classes confer hepatic benefit or risk.

Additional therapeutic approaches under investigation include fecal microbiota transplantation (FMT), which may modulate the proinflammatory state associated with chronic liver disease. By reshaping the gut microbiota and improving gut barrier integrity, FMT can reduce systemic and hepatic inflammation and may attenuate fibrosis progression, ultimately leading to better clinical outcomes. However, its role in MASLD requires further investigation [50].

Several limitations of our study should be acknowledged. The cross-sectional design precludes causal inference, and the control group differed from patient groups in sex distribution and metabolic profile, which may have influenced comparisons despite statistical adjustment. Self-reported alcohol consumption may underestimate actual intake; however, this potential bias would be expected to affect both groups similarly. PASI scores were obtained at a single time point, limiting the assessment of cumulative inflammatory burden. Lifestyle factors such as diet and physical activity were also not systematically captured. Although all participants underwent abdominal ultrasound as part of the routine clinical assessment and study protocol, ultrasound findings were not prospectively collected as predefined study variables. Consequently, they were not included in the statistical analyses or incorporated into the predefined study outcomes, which were based on transient elastography-derived parameters (CAP and liver stiffness measurements) together with non-invasive fibrosis scores.

MASLD is increasingly recognized as a common condition that shares pathophysiologic mechanisms with other metabolic disorders frequently observed in PsD. These overlaps suggest that hepatic assessment may have a role within the broader evaluation of PsD, particularly in individuals with metabolic risk factors. Further research may help determine whether inflammatory mechanisms in PsD influence fibrosis progression, which could ultimately refine monitoring strategies and support more tailored patient management.

## Figures and Tables

**Table 1:** Characteristics of patients with psoriasis, psoriatic arthritis and healthy subjects.

	Psoriasis (*n* = 101)	Psoriatic Arthritis (*n* = 127)	Healthy Subjects (*n* = 102)	*p*
Men, *n* (%)	64 (63.4%)	65 (51.2%)	28 (27.5%)	<0.001
Women, *n* (%)	37 (36.6%)	62 (48.8%)	74 (72.5%)	<0.001
Age (years), mean ± SD	57.8 ± 12.7	59.7 ± 12.2	53.3 ± 13.7	0.25
Cardiovascular risk factors				
Diabetes mellitus, *n* (%)	14 (13.9%)	16 (12.6%)	3 (2.9%)	0.016
Glycemia (mg/dL), mean ± SD	98.9 ± 25	98.4 ± 21.7	92.6 ± 17	0.067
Hypertension, *n* (%)	45 (44.6%)	37 (29.1%)	7 (6.9%)	<0.001
Dyslipidemia, *n* (%)	45 (44.6%)	57 (44.9%)	8 (7.8%)	<0.001
LDL-C (mg/dL), mean ± SD	116.3 ± 33.9	123.3 ± 34.3	119.9 ± 34.5	0.047
TG (mg/dL), mean ± SD	124.1 ± 61.8	121.8 ± 68.4	81.5 ± 43.7	<0.001
BMI (kg/m^2^), mean ± SD	29.2 ± 6.03	28.04 ± 4.7	25.12 ± 4.03	<0.001
Hyperuricemia, *n* (%)	6 (5.9%)	10 (7.9%)	5 (4.9%)	0.643
Transaminases				
ALT (U/L), mean ± SD	26.6 ± 13.6	27.1 ± 12.4	20.5 ± 9.4	<0.001
AST (U/L), mean ± SD	23.1 ± 8.9	23.4 ± 7.6	20.6 ± 6.0	0.015

Abbreviations: ALT = alanine aminotransferase, AST = aspartate aminotransferase, BMI = body mass index, LDL-C = low density lipoprotein-cholesterol, and TG = triglycerides.

**Table 2:** Results of the FibroScan^®^ and the scoring tools of patients with psoriasis, psoriatic arthritis and healthy subjects.

	Psoriasis (*n* = 101)	Psoriatic Arthritis (*n* = 127)	Healthy Subjects (*n* = 102)	*p*
FibroScan^®^				
Fibrosis (kPa), mean ± SD	6.01 ± 3.4	5.1 ± 1.7	4.9 ± 2.08	0.006
F0, *n* (%)	76 (75.2%)	103 (81.1%)	92 (90.2%)	
F2, *n* (%)	13 (12.95%)	9 (7.1%)	3 (2.9%)	
F3, *n* (%)	3 (3%)	7 (5.5%)	2 (2%)	
F4, *n* (%)	7 (6.9%)	1 (0.8%)	4 (3.9%)	
CAP (dB/m), mean ± SD	270.49 ± 53.7	257 ± 52.54	229 ± 47.51	<0.001
S0, *n* (%)	28 (27.7%)	48 (37.8%)	62 (60.8%)	
S1, *n* (%)	16 (15.8%)	18 (14.2%)	13 (12.7%)	
S2, *n* (%)	22 (21.8%)	22 (17.3%)	14 (13.7%)	
S3, *n* (%)	33 (32.7%)	32 (25.2%)	12 (11.8%)	
Scoring tools				
FIB-4, mean ± SD	1.22 ± 0.56	1.22 ± 0.58	1.04 ± 0.48	0.024
NFS, mean ± SD	−1.67 ± 1.3	−1.89 ± 1.2	−2.3 ± 1	<0.001
APRI, mean ± SD	0.28 ± 0.12	0.3 ± 0.15	0.26 ± 0.09	0.075
HFS, mean ± SD	0.06 ± 0.11	0.03 ± 0.05	0.01 ± 0.03	0.006

Abbreviations: CAP = controlled attenuation parameter, F = fibrosis, FIB-4 = fibrosis-4, APRI = the aspartate aminotransferase-to-platelet ratio index, HFS = the hepamet fibrosis score, NFS = the NAFLD score, S = steatosis.

## Data Availability

The original contributions presented in this study are included in the article. Further inquiries can be directed to the corresponding author.
